# Whole-tissue 3D imaging reveals intra-adipose sympathetic plasticity regulated by NGF-TrkA signal in cold-induced beiging

**DOI:** 10.1007/s13238-018-0528-5

**Published:** 2018-03-27

**Authors:** Ying Cao, Huanhuan Wang, Wenwen Zeng

**Affiliations:** 1 Center for Life Sciences, Tsinghua University, Beijing, 100084 China; 2 Institute for Immunology and School of Medicine, Tsinghua University, Beijing, 100084 China; 3 Beijing Key Laboratory for Immunological Research on Chronic Diseases, Beijing, 100084 China; 4 Academy for Advanced Interdisciplinary Studies, Peking University, Beijing, 100871 China; 5 School of Life Sciences, Peking University, Beijing, 100871 China

**Keywords:** sympathetic plasticity, NGF, TrkA receptor, cold-induced beiging, whole-tissue 3D imaging

## Abstract

Sympathetic arborizations act as the essential efferent signals in regulating the metabolism of peripheral organs including white adipose tissues (WAT). However, whether these local neural structures would be of plastic nature, and how such plasticity might participate in specific metabolic events of WAT, remains largely uncharacterized. In this study, we exploit the new volume fluorescence-imaging technique to observe the significant, and also reversible, plasticity of intra-adipose sympathetic arborizations in mouse inguinal WAT in response to cold challenge. We demonstrate that this sympathetic plasticity depends on the cold-elicited signal of nerve growth factor (NGF) and TrkA receptor. Blockage of NGF or TrkA signaling suppresses intra-adipose sympathetic plasticity, and moreover, the cold-induced beiging process of WAT. Furthermore, we show that NGF expression in WAT depends on the catecholamine signal in cold challenge. We therefore reveal the key physiological relevance, together with the regulatory mechanism, of intra-adipose sympathetic plasticity in the WAT metabolism.

**Electronic supplementary material:**

The online version of this article (10.1007/s13238-018-0528-5) contains supplementary material, which is available to authorized users.

## Introduction

The central nervous system exerts the indispensable control over maintenance of the metabolic homeostasis. Brain circuits, together with their distinct neuronal populations, underlying such neural regulation have been subject to extensive research. It has now been broadly accepted that malfunction of the neural regulation of metabolism could lead to obesity, type 2 diabetes and other profound metabolic disorders (Friedman and Halaas, 1998; Gautron et al., 2015; Myers and Olson, 2012).

As one of the emerging focuses in the field, studies have begun to investigate how efferent signals from the central nervous system reach out to regulate the metabolism of peripheral organs, e.g., white adipose tissues (WAT). WAT are known as the key energy-storage depots as well as the important hormone-producing organ, whose essential roles in the energy homeostasis are widely recognized. Anatomical distribution and physiological function of local neural inputs, particularly sympathetic inputs, in WAT have been explored to understand the mechanism of neural regulation of the WAT metabolism (Bamshad et al., 1998; Nguyen et al., 2014; Youngstrom and Bartness, 1995, 1998; Zeng et al., 2015). For instance, sympathetic fibers can form synapse-like structures onto adipocytes in WAT, and destruction of these sympathetic innervations inhibited the leptin-stimulated lipolysis of WAT (Zeng et al., 2015). In addition, accumulating research efforts have revealed that WAT can undergo the beiging (or browning) process, i.e., appearance of Ucp1-positive multilocular adipocytes (or beige cells), under certain physiological conditions such as cold exposure. This metabolic event of WAT results in enhanced energy expenditure, and therefore, has increasingly gained attentions for its potential application in therapeutic prevention and treatment of obesity and type 2 diabetes (Giordano et al., 2016; Harms and Seale, 2013; Kajimura et al., 2015; Peirce et al., 2014; Rosen and Spiegelman, 2014). Adding to the key function of local sympathetic inputs in the WAT metabolism, it has been recently reported that the dense network of intra-adipose sympathetic arborizations in WAT is required for the cold-induced beiging process (Jiang et al., 2017).

Despite those important progresses, our understanding of neural regulation of the WAT metabolism still remains incomplete. In particular, sympathetic arborizations in the peripheral organs have been generally viewed as being static structures under physiological condition. Whether local sympathetic arborizations are in fact of the dynamic nature, i.e., plasticity, in response to certain metabolic stimuli is largely uncharacterized. A previous report showed with the conventional immunohistochemistry that numbers of the sympathetic fibers in WAT would increase following cold exposure (Vitali et al., 2012). However, the physiological relevance, together with the regulatory mechanism, underlying potential intra-adipose sympathetic plasticity is unknown.

In this report, we exploit the new volume fluorescence-imaging technique to observe the significant, and also reversible, plasticity of sympathetic arborizations in mouse inguinal WAT in response to cold challenge. We demonstrate that this sympathetic plasticity is regulated by the cold-elicited signal of nerve growth factor (NGF) and TrkA receptor. Blockage of NGF-TrkA signaling suppresses intra-adipose sympathetic plasticity, and importantly, inhibits the cold-induced beiging process of WAT. We further show that cold-elicited NGF expression in WAT depends on the catecholamine signal during cold challenge. This study has therefore documented the key physiological function as well as the molecular mechanism of local sympathetic plasticity in WAT. These findings would provide important insights to our understanding of neural control of the peripheral metabolism under physiological and disease conditions.

## Results

### Plasticity of intra-adipose sympathetic arborizations in response to cold challenge

**Figure 1:**
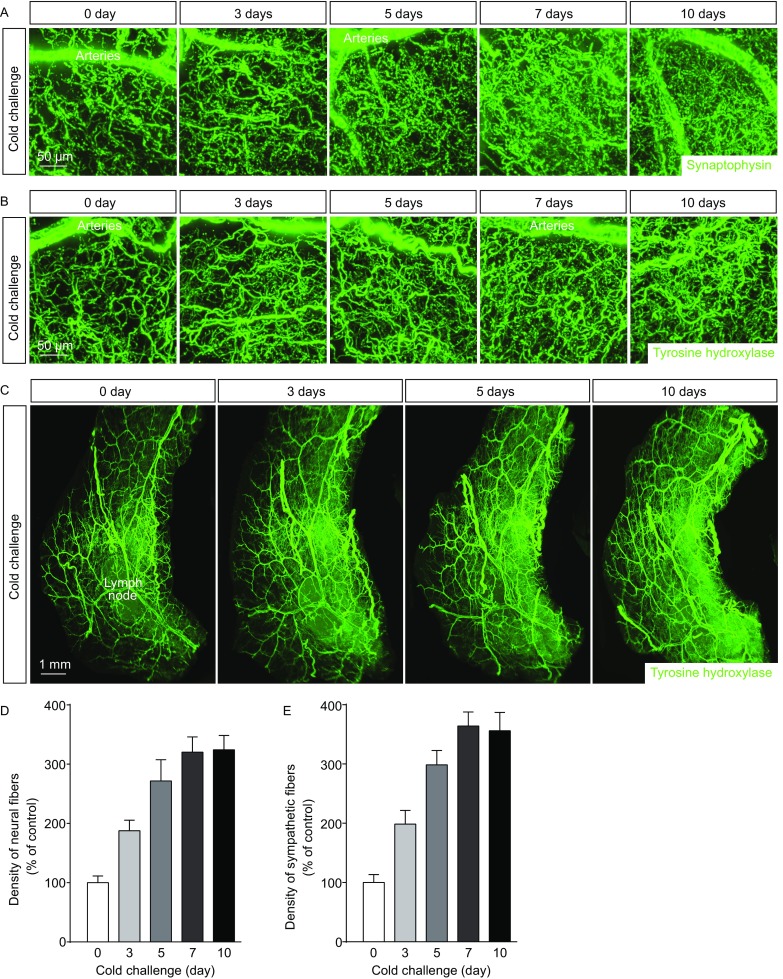
**Intra-adipose sympathetic plasticity in response to cold challenge**. (A–E) The wildtype mice were subject to cold challenge. (A) Representative 3D projections of iWAT harvested at indicated time points, immunolabeled by anti-synaptophysin, and imaged at 12.6× magnification on the lightsheet microscope. (B and C) Representative 3D projections of iWAT harvested at indicated time points, immunolabeled by anti-tyrosine hydroxylase, and imaged at 12.6× (B) or 1.26× (C) magnification on the lightsheet microscope. (D) Density of the neural fibers in iWAT immunolabeled by anti-synaptophysin was quantified. *n* = 4, mean ± SEM. (E) Density of the sympathetic fibers in iWAT immunolabeled by anti-tyrosine hydroxylase was quantified. *n* = 5, mean ± SEM

We set out to examine the sympathetic arborizations in mouse inguinal WAT (iWAT) in response to cold challenge (4 °C). We exploited the new volume fluorescence-imaging technique that we have recently established for adipose tissues, which enables us to easily visualize and quantify intra-adipose neural fibers on the whole-tissue level (Jiang et al., 2017). iWAT was processed through the whole-mount immunolabeling of intended epitopes, and then subjected to the optical clearing procedure for imaging on the lightsheet microscope. We firstly assessed density of the total neural fibers in iWAT of the wildtype mice by the volume fluorescence-imaging of anti-synaptophysin, an integral protein on pre-synaptic vesicles and a pan-marker for neural structures. The significant increase of total neural fibers in iWAT could be observed as early as 3 days after cold challenge, and their density reached a plateau between 7 to 10 days (Fig. 1A and 1D). We have recently shown that the neural fibers in iWAT are predominantly sympathetic (Jiang et al., 2017). To determine whether such cold-induced increase of neural density in iWAT would reflect the plastic change of sympathetic arborizations, iWAT was also visualized by the volume fluorescence-imaging of anti-tyrosine hydroxylase, a specific marker for sympathetic fibers. Indeed, density of the sympathetic fibers showed the significant increase as early as 3 days after cold challenge (Fig. 1B and 1E, and Movies S1 and S2). Importantly, this plastic change of sympathetic arborizations in iWAT correlated with up-regulation of the beiging-related genes including *Ucp1*, *Dio2*, *Cidea* and *Pgc1a* (Fig. S1A). Of note, there was no significant alteration of the tissue size, as well as the general anatomy of sympathetic innervations, of the cold-challenged iWAT as visualized by the volume fluorescence-imaging at low magnification (Fig. 1C).

**Figure 2:**
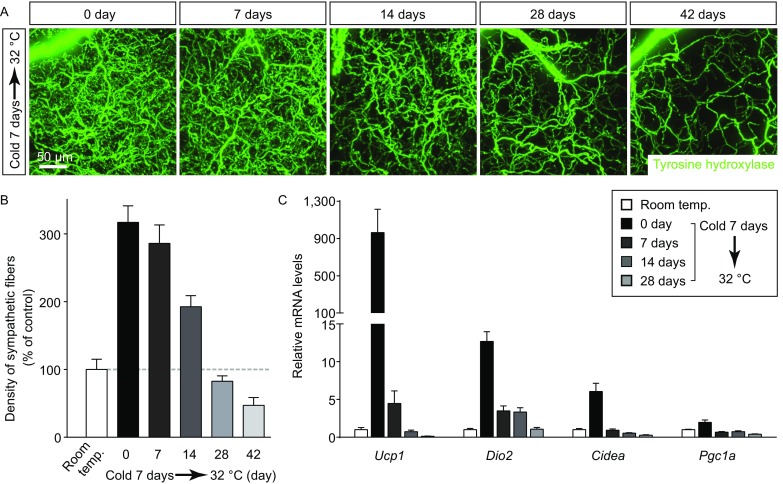
**Cold-induced intra-adipose sympathetic plasticity is reversible**. The wildtype mice were subject to cold challenge, and then recovered at thermal-neutral condition (32 °C). (A) Representative 3D projections of iWAT harvested at indicated time points, immunolabeled by anti-tyrosine hydroxylase, and imaged at 12.6× magnification on the lightsheet microscope. (B) Density of the sympathetic fibers in iWAT was quantified. *n* = 5, mean ± SEM. (C) Expression levels of the beiging-related genes were determined at indicated time points by the qPCR analysis. *n* = 5, mean ± SEM

We next explored whether this cold-induced plasticity of sympathetic arborizations in iWAT would be reversible. The wildtype mice were subject to cold challenge for 7 days, and then recovered at thermal-neutral condition (32 °C) for up to 6 weeks. Density of the sympathetic arborizations in iWAT underwent the significant decrease around 2 weeks under thermal-neutral condition, and reached the baseline level at 4 weeks (Fig. 2A and 2B), as assessed by the volume fluorescence-imaging of anti-tyrosine hydroxylase. Interestingly, the sympathetic density could further decline to about 50% of the baseline level at 6 weeks under thermal-neutral condition (Fig. 2A and 2B). In addition, such reversal of intra-adipose sympathetic arborizations correlated with down-regulation of the beiging-related genes *Ucp1*, *Dio2*, *Cidea* and *Pgc1a* (Fig. 2C). These results have together demonstrated that intra-adipose sympathetic arborizations have the significant, and also reversible, plasticity in response to cold challenge, which might be involved in the cold-induced beiging process of WAT.

### Intra-adipose sympathetic plasticity regulated by the NGF-TrkA signal is required for cold-induced beiging

**Figure 3:**
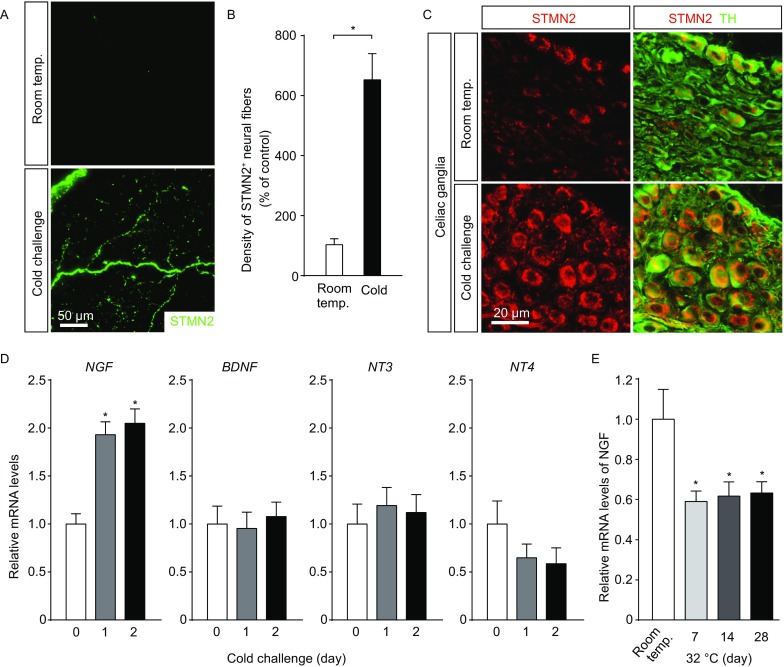
**Cold-elicited NGF expression for intra-adipose sympathetic plasticity**. (A–D) The wildtype mice were subject to cold challenge. (A and B) iWAT was processed for the whole-mount immunolabeling of STMN2. (A) Representative 3D projections of iWAT imaged at 12.6× magnification on the lightsheet microscope. (B) Density of the STMN2-positive neural fibers was quantified. *n* = 4, mean ± SEM, *P <  0.01. (C) STMN2 expression in sympathetic neurons of the celiac ganglia was examined by conventional immunostaining. (D) Expression levels of the neurotrophin genes in iWAT were determined by the qPCR analysis. *n* = 4, mean ± SEM, *P < 0.01. (E) The wildtype mice were subject to thermal-neutral condition (32 °C), and expression levels of *NGF* in iWAT were determined by the qPCR analysis. *n* = 4, mean ± SEM, *P < 0.01

We then sought out to elucidate the physiological relevance, together with the regulatory mechanism, of intra-adipose sympathetic plasticity in response to cold challenge. The volume fluorescence-imaging revealed that there was the significant up-regulation of STMN2 expression (also known as SCG10), a specific marker for axon outgrowth (Grenningloh et al., 2004; Mason et al., 2002), in iWAT after cold challenge (Fig. 3A and 3B). In addition, we recently showed that the celiac ganglia contribute to the sympathetic innervations in mouse iWAT (Jiang et al., 2017). Accordingly, STMN2-positive sympathetic neurons in the celiac ganglia also dramatically increased after cold challenge (Fig. 3C). These results have suggested that an active process of sympathetic axon outgrowth is promoted in the cold-challenged iWAT.

Axon outgrowth has been known to be under regulation of the neurotrophic factors (or neurotrophins) (Bothwell, 1995; Raffioni et al., 1993; Zweifel et al., 2005). We therefore profiled expression levels of the neurotrophin genes in iWAT of the wildtype mice exposed to cold challenge, and observed that *NGF* expression showed the significant up-regulation as early as 24 h after cold challenge (Fig. 3D). In contrast, expression levels of other neurotrophin genes *BDNF*, *NT3*, and *NT4* exhibited no increase in iWAT in response to cold challenge (Fig. 3D). Also, it is intriguing to note that expression levels of *NGF* decreased significantly in iWAT of the wildtype mice shifted from room temperature to 32 °C (Fig. 3E), which appeared correlating with the declined density of sympathetic arborizations in iWAT under this thermal-neutral condition (Fig. 2A and 2B).

**Figure 4:**
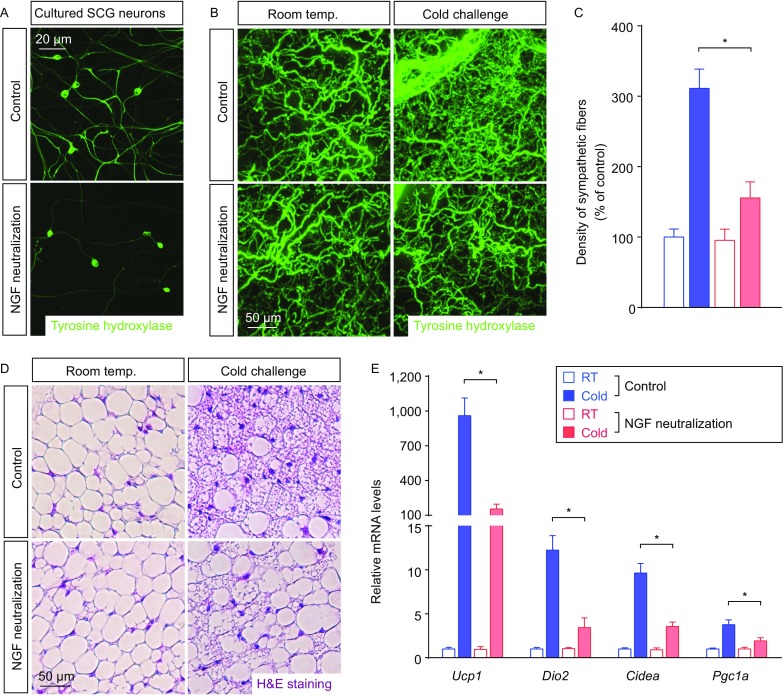
**Intra-adipose sympathetic plasticity regulated by NGF is essential for cold-induced beiging process**. (A) Representative images of cultured sympathetic SCG neurons treated with NGF-neutralizing antibody or control IgG. (B–E) The wildtype mice administrated with NGF-neutralizing antibody or control IgG were subject to cold challenge. (B) iWAT was processed for the volume fluorescence-imaging of anti-tyrosine hydroxylase. Representative 3D projections of iWAT imaged at 12.6× magnification on the lightsheet microscope. (C) Density of the sympathetic fibers in iWAT was quantified. *n* = 5, mean ± SEM, *P < 0.01. (D) Appearance of multilocular beige cells in iWAT was examined by H&E staining. (E) Expression levels of the beiging-related genes in iWAT were determined by the qPCR analysis. *n* = 5, mean ± SEM, *P < 0.01

As one of the central components in neural development, genetic deletion of *NGF* results in severe developmental defects of the nervous systems, including the sympathetic system (Crowley et al., 1994). To explore the potential function of NGF in intra-adipose sympathetic plasticity without the developmental complications, we set out to acutely block the NGF signal by NGF neutralization. NGF-neutralizing antibody could strongly suppress the NGF-stimulated axon outgrowth of cultured sympathetic neurons of the superior cervical ganglia (SCG), demonstrating the efficacy of this neutralization strategy (Fig. 4A). The wildtype mice were intravenously administrated with NGF-neutralizing antibody (i.e., NGF-blocked mice) or isotype control IgG. The volume fluorescence-imaging revealed no detectable alteration of the tissue size and the general anatomy of sympathetic innervations (Fig. S2A), as well as density of the sympathetic arborizations (Fig. 4B and 4C), of iWAT of the NGF-blocked mice vs. control mice maintained at room temperature for up to 1 week. However, while density of the sympathetic arborizations increased significantly in control mice after cold challenge, such intra-adipose sympathetic plasticity was largely inhibited in the NGF-blocked mice (Fig. 4B and 4C). More importantly, this inhibition of sympathetic plasticity led to the diminished beiging process of iWAT, as assessed by the histochemical examination of multilocular beige cells (Fig. 4D) and the qPCR analysis of beiging-related genes *Ucp1*, *Dio2*, *Cidea* and *Pgc1a* (Fig. 4E). These results have demonstrated that NGF regulates intra-adipose sympathetic plasticity in response to cold challenge, which is critical for the cold-induced beiging process.

**Figure 5:**
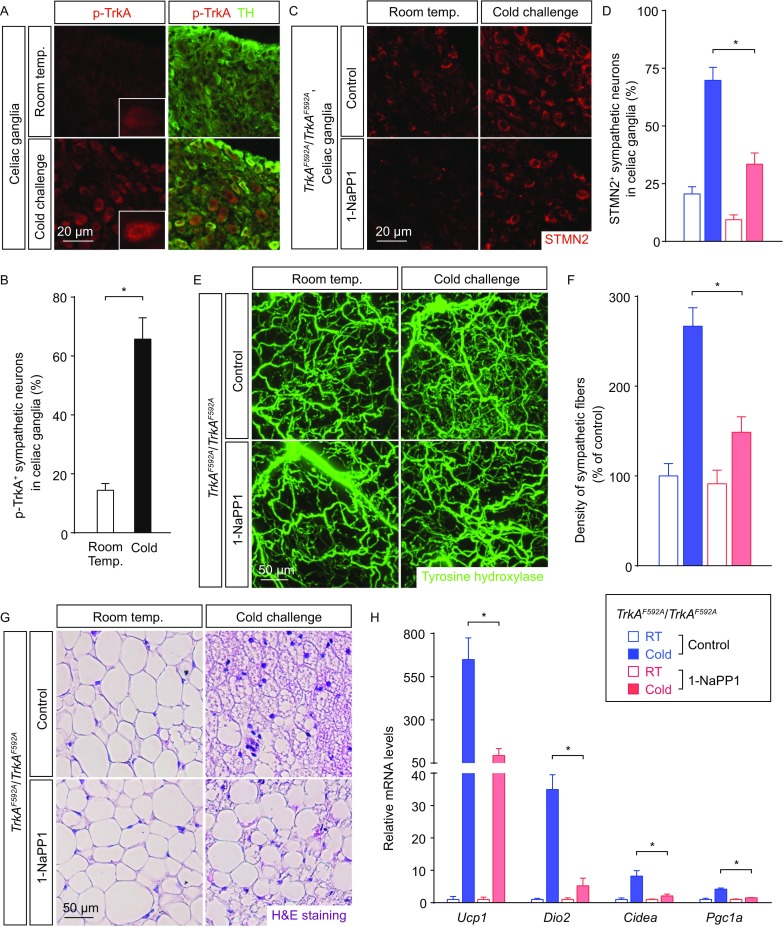
**Intra-adipose sympathetic plasticity regulated by the TrkA signal is required for cold-induced beiging process**. (A and B) The wildtype mice were subject to cold challenge. (A) Activation of the TrkA signal in sympathetic neurons of the celiac ganglia was examined by conventional immunostaining of anti-p-TrkA. Insets, representative zoom-in views of vesicular p-TrkA localization. (B) p-TrkA-positive sympathetic neurons of the celiac ganglia were quantified. *n* = 4, mean ± SEM, *P < 0.01. (C–H) *TrkA*^*F592A*/*F592A*^ mice daily-treated with 1-NaPP1 or vehicle control were subject to cold challenge. (C) STMN2 expression in sympathetic neurons of the celiac ganglia was examined by conventional immunostaining. (D) STMN2-positive sympathetic neurons of the celiac ganglia were quantified. *n* = 4, mean ± SEM, *P < 0.01. (E) iWAT was processed for the volume fluorescence-imaging of anti-tyrosine hydroxylase. Representative 3D projections of iWAT imaged at 12.6× magnification on the lightsheet microscope. (F) Density of the sympathetic fibers in iWAT was quantified. *n* = 5, mean ± SEM, *P < 0.01. (G) Appearance of multilocular beige cells in iWAT was examined by H&E staining. (H) Expression levels of the beiging-related genes in iWAT were determined by the qPCR analysis. *n* = 5, mean ± SEM, *P < 0.01

Sympathetic neurons in the celiac ganglia that innervate iWAT predominantly express TrkA, the high-affinity receptor for NGF (Bothwell, 1995; Raffioni et al., 1993; Zweifel et al., 2005). Consistent with the up-regulated NGF expression in iWAT after cold challenge, significant activation of the TrkA signal was observed in sympathetic neurons of the celiac ganglia, as assessed by p-TrkA immunostaining (Fig. 5A and 5B). Of note, p-TrkA exhibited the vesicular localization in neuronal cell bodies (Fig. 5A), reflecting the retrograde vesicular transport of TrkA receptor (Zweifel et al., 2005). Previous studies have documented the essential roles of TrkA receptor in neural development, and genetic deletion of *TrkA* severely impairs establishment of the sympathetic system (Smeyne et al., 1994). Therefore, to investigate the TrkA signal in intra-adipose sympathetic plasticity without perturbing the normal sympathetic development, we exploited the chemical-genetic approach of *TrkA*^*F592A*/*F592A*^ mice. In this knock-in mouse line, Phe592 residue at the ATP-binding pocket of TrkA has been replaced by Ala, enabling the efficient inhibition of TrkA^F592A^ activity by the chemical 1-NaPP1, which otherwise has little effect on the wildtype version of TrkA protein (Chen et al., 2005). *TrkA*^*F592A*/*F592A*^ mice were treated daily with 1-NaPP1 or vehicle control. The volume fluorescence-imaging revealed no significant alteration of the tissue size and the general anatomy of sympathetic innervations (Fig. S3A), or density of the sympathetic arborizations (Fig. 5E and 5F), of iWAT of the 1-NaPP1-treated vs. vehicle-treated *TrkA*^*F592A*/*F592A*^ mice maintained at room temperature for up to 1 week. However, when the mice were subject to cold challenge, axon outgrowth response of sympathetic neurons in the celiac ganglia was significantly inhibited by 1-NaPP1, as assessed by anti-STMN2 immunostaining (Fig. 5C and 5D). Accordingly, the sympathetic plasticity in iWAT was strongly suppressed in the 1-NaPP1-treated *TrkA*^*F592A*/*F592A*^ mice, compared to that normally occurring in the vehicle-treated *TrkA*^*F592A*/*F592A*^ mice (Fig. 5E and 5F). Moreover, such inhibition of intra-adipose sympathetic plasticity suppressed the beiging process of iWAT in the 1-NaPP1-treated *TrkA*^*F592A*/*F592A*^ mice, as determined by the histochemical examination of multilocular beige cells (Fig. 5G) and the qPCR analysis of beiging-related genes (Fig. 5H). Of importance, 1-NaPP1 treatment of the cold-challenged wildtype mice had no effect on the sympathetic plasticity in iWAT (Fig. S3B), appearance of multilocular beige cells (Fig. S3C), or expression of the beiging-related genes (Fig. S3D), confirming the exquisite specificity of this chemical-genetic strategy. Taken together, the results have shown that intra-adipose sympathetic plasticity regulated by the NGF-TrkA signal has the key involvement in the cold-induced beiging process.

### The catecholamine signal regulates NGF expression for intra-adipose sympathetic plasticity

**Figure 6:**
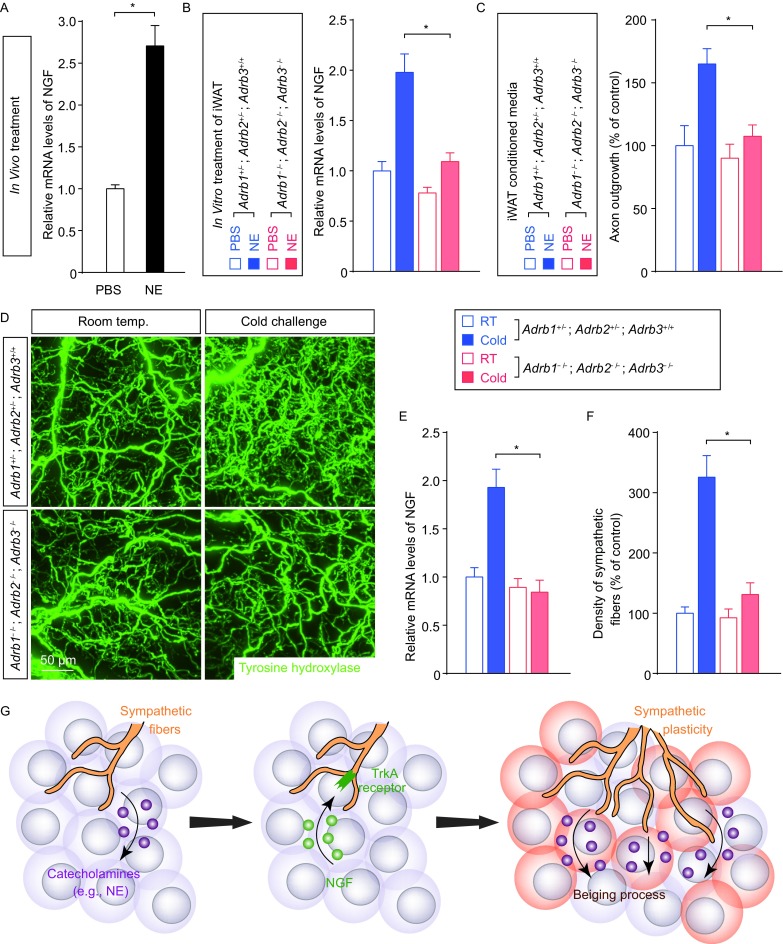
**Cold-elicited NGF expression in WAT depends on the catecholamine signal**. (A) The wildtype mice maintained at room temperature were treated with norepinephrine (NE) or vehicle control. Expression levels of *NGF* in iWAT were determined by the qPCR analysis. *n* = 5, mean ± SEM, *P < 0.01. (B and C) iWAT of *Adrb1*^−/−^; *Adrb2*^−/−^; *Adrb3*^−/−^mice or control mice (*Adrb1*^+/−^; *Adrb2*^+/−^; *Adrb3*^+/+^) was *in vitro* treated with 100 μmol/L norepinephrine (NE) or control PBS. (B) Expression levels of *NGF* in iWAT were determined by the qPCR analysis. *n* = 4, mean ± SEM, *P < 0.01. (C) The conditioned media of iWAT were collected and administrated onto cultured sympathetic neurons. Lengths of the axon outgrowth were quantified. *n* = 4, mean ± SEM, *P < 0.01. (D–F) *Adrb1*^−/−^; *Adrb2*^−/−^; *Adrb3*^−/−^ mice or control mice (*Adrb1*^+/−^; *Adrb2*^+/−^; *Adrb3*^+/+^) were subject to cold challenge. (D) iWAT was processed for the volume fluorescence-imaging of anti-tyrosine hydroxylase. Representative 3D projections of iWAT imaged at 12.6× magnification on the lightsheet microscope. (E) Expression levels of *NGF* in iWAT were determined by the qPCR analysis. *n* = 4, mean ± SEM, *P < 0.01. (F) Density of the sympathetic fibers in iWAT was quantified. *n* = 4, mean ± SEM, *P < 0.01. (G) Diagram of the key involvement of intra-adipose sympathetic plasticity regulated by the NGF-TrkA signal in the cold-induced beiging process of WAT

We next examined the mechanism regulating NGF expression in response to cold challenge. Studies in the field have documented the central role of catecholamines (i.e., epinephrine and norepinephrine) in the beiging process. In particular, genetic deletion of key enzymes in the catecholamine synthesis or β-adrenergic receptors for catecholamines could strongly suppress the cold-induced beiging process (Barbatelli et al., 2010; Fischer et al., 2017; Jiang et al., 2017; Jimenez et al., 2003; Qiu et al., 2014). We therefore explored whether the catecholamine signal would be involved in the cold-elicited NGF expression. We observed that treatment with norepinephrine (NE) efficiently increased the expression levels of *NGF* in iWAT of the wildtype mice maintained at room temperature (Fig. 6A). Moreover, the *in vitro* treatment of acutely-dissected iWAT of control mice (i.e., *Adrb1*^+/−^;* Adrb2*^+/−^;* Adrb3*^+/+^) with NE enhanced the *NGF* expression levels, but such effect was abolished in iWAT of *Adrb1*^−/−^;* Adrb2*^−/−^;* Adrb3*^−/−^ mice (Fig. 6B), in which all the three genes of β-adrenergic receptors are deleted. To examine whether NE-treatment would stimulate the production of functional NGF protein by iWAT, we tested the conditioned media of *in vitro* treated iWAT on cultured sympathetic neurons. Indeed, the conditioned media of NE-treated iWAT of *Adrb1*^+/−^;* Adrb2*^+/−^;* Adrb3*^+/+^ control mice, but not *Adrb1*^−/−^;* Adrb2*^−/−^;* Adrb3*^−/−^ mice, exhibited the increased ability to promote the sympathetic axon outgrowth *in vitro* (Fig. 6C and Fig. S4A).

To further determine the regulatory function of catecholamine signal in intra-adipose sympathetic plasticity, *Adrb1*^−/−^;* Adrb2*^−/−^;* Adrb3*^−/−^ mice were examined in the cold-challenge condition. Supporting the *in vitro* observation, the cold-elicited *NGF* up-regulation was absent in iWAT of *Adrb1*^−/−^;* Adrb2*^−/−^;* Adrb3*^−/−^ mice, compared to that normally occurring in iWAT of *Adrb1*^+/−^;* Adrb2*^+/−^;* Adrb3*^+/+^ control mice (Fig. 6E). More importantly, in line with this blunted *NGF* response, density of the sympathetic arborizations remained unchanged in *Adrb1*^−/−^;* Adrb2*^−/−^;* Adrb3*^−/−^ mice after cold challenge as assessed by the volume fluorescence-imaging of anti-tyrosine hydroxylase (Fig. 6D and 6F), suggesting the complete loss of sympathetic plasticity with this simultaneous deletion of β-adrenergic receptors. The results have together revealed that the catecholamine signal regulates NGF expression for intra-adipose sympathetic plasticity in response to cold challenge.

## Discussion

In summary, aided by the new volume fluorescence-imaging technique, our study has revealed that intra-adipose sympathetic plasticity, regulated by the cold-elicited NGF-TrkA signal, exerts a key role in the beiging process of WAT. To our knowledge, this research work represents among the first examples demonstrating the physiological relevance, together with the molecular mechanism, of local sympathetic plasticity in neural regulation of the peripheral metabolism.

Intriguingly, the catecholamine signal derived from the local sympathetic arborizations in WAT promotes NGF expression in response to cold challenge. This cold-elicited NGF in turn stimulates the sympathetic axon outgrowth. This plastic change appears to have evolved as a positive-feedback mechanism of locally enhancing the sympathetic efferent outputs to ensure effectiveness of the beiging process (Fig. 6G). Our findings have therefore implicated an additional layer of neural regulation in the WAT metabolism, which involves the crosstalk between the neural-metabolic systems. However, the cellular source(s) of NGF protein in WAT remains to be determined, and the volume fluorescence-imaging technique would serve to provide the important clues. In addition, the molecular mechanism underlying the catecholamine-stimulated NGF expression requires further detailed characterization, which likely engages the protein kinase A (PKA) signal downstream of β-adrenergic receptors. The future research efforts have been warranted to explore these important questions.

Our current work has been focused on the sympathetic plasticity in mouse inguinal WAT. Importantly, a recent study in the field has systematically characterized the adipose tissues in mice, revealing the heterogeneity of beiging capacity among different depots (Zhang et al., 2018). The volume fluorescence-imaging technique is readily applicable to WAT and BAT (Jiang et al., 2017), which makes it possible to explore whether the NGF/TrkA-dictated sympathetic plasticity might also occur in other adipose depots, e.g., epididymal WAT or BAT, in response to cold challenge. Of note, our findings that density of the sympathetic arborizations increased in the cold-challenged iWAT are in accordance with the previous study done with the conventional immunohistochemistry (Vitali et al., 2012). However, our results are in disagreement with a recent report suggesting that the sympathetic density remained unchanged in iWAT after cold challenge (Chi et al., 2018). Such discrepancy might be due to the different procedure of whole-mount immunostaining used in this recent report (Chi et al., 2018).

This current study would provide new insights to neural control of the peripheral metabolism not only under physiological condition but also in metabolic disease. In fact, our previous work has reported that density of the sympathetic arborizations in WAT dramatically decreased in the obese condition, e.g., in the high-fat diet-fed mice or *ob*/*ob* mice (Jiang et al., 2017). Whether this phenomenon could reflect dys-regulation of intra-adipose sympathetic plasticity, and whether impairment of the NGF-TrkA signal would be causative, then needs to be determined. Conversely, harnessing this sympathetic plasticity to regenerate the intra-adipose arborizations afflicted in metabolic disorders might provide a novel entry point for therapeutic strategy to restore the local sympathetic efferent outputs, and as the result, the metabolic homeostasis of WAT.

## Materials and methods

### Animal information

All the experimental procedures in mice were performed in compliance with the protocol approved by the Institutional Animal Care and Use Committee (IACUC) of Tsinghua University.

Animals were maintained on the 12-h light/12-h dark cycles with the chow diet and water available *ad libitum*. Mice utilized in the experiments were females at the age of 2 to 4 months. Wildtype C57BL/6 mice were purchased from the Charles River International. *TrkA*^*F592A*/*F592A*^ (JAX 022362, RRID:IMSR_JAX:022362), *Adrb1*^−/−^;*Adrb2*^−/−^ (JAX 003810, RRID:IMSR_JAX:003810), and *Adrb3*^−/−^ (JAX 006402, RRID:IMSR_JAX:006402) were from the Jackson Laboratory, and in-house bred to produce the littermates, which were randomly assigned to experimental groups.

The mice of indicated genotypes were transferred from room temperature (22–23 °C) to 4 °C for cold challenge, or to 32 °C for thermal-neutral condition. For the experiments of NGF neutralization, NGF-neutralizing antibody or isotype control IgG was administrated to the wildtype mice at 10 mg/kg of body weight via intravenous injection. For the experiments of chemical-genetic inhibition of TrkA, 1-NaPP1 was formulated in DMSO/Kolliphor-EL/5% sucrose (1:3:6) and administrated to *TrkA*^*F592A*/*F592A*^ mice or the wildtype mice at 10 mg/kg of body weight via oral gavage every 24 h. For the norepinephrine treatment, norepinephrine was administrated to the wildtype mice at 10 mg/kg of body weight via intraperitoneal injection.

### Antibodies

Primary antibodies used for immunolabeling were rabbit anti-Tyrosine hydroxylase (Millipore, #AB152, RRID:AB_390204), chicken anti-Tyrosine hydroxylase (Millipore, #AB9702, RRID:AB_570923), rabbit anti-Synaptophysin (Invitrogen, #18-0130, RRID:AB_10836766), rabbit anti-STMN2 (Novus Biologicals, #NBP1-49461, RRID:AB_10011568), and rabbit anti-p-TrkA (Cell Signaling, #4168, RRID:AB_10620952). In addition, Alexa dye-conjugated secondary antibodies were from Life Technologies.

NGF-neutralizing antibody (mouse IgG1) was from Thermo Fisher Scientific (#MA1-12347, RRID:AB_1077262), and mouse IgG1 isotype control was from BioXCell (#BE0083, RRID:AB_1107784).

### Tissue processing

To determine expression levels of the genes, iWAT was acutely dissected from the mice at indicated time points after treatment. The total RNAs were extracted by RNeasy Mini Lipid Tissue Kit (Qiagen), and processed for SYBR Green (Thermo Fisher Scientific) qPCR analysis. To examine appearance of the cold-induced beige adipocytes, iWAT was fixed in PBS/1% PFA at 4 °C overnight, and processed for paraffin-sectioning and H&E (hematoxylin and eosin) staining.

To examine sympathetic neurons of the celiac ganglia, the ganglia were acutely dissected from the mice of indicated conditions. The tissues were fixed in PBS/1% PFA at 4 °C overnight, and processed for cryosectioning. The sections were immunostained with indicated primary antibodies and corresponding Alexa dye-conjugated secondary antibodies, and imaged by the fluorescence microscopy.

### Volume fluorescence-imaging

The volume fluorescence-imaging procedure of WAT was performed as recently reported (Jiang et al., 2017). The mice of indicated conditions were anesthetized, and perfused with PBS containing 10 μg/mL heparin (Sigma). iWAT was dissected out, and fixed in PBS/1% PFA/10% sucrose at 4 °C overnight. The tissues were washed with PBS for 1 h three times, and the attached connective tissues were removed under a dissecting microscope. The tissues were dehydrated at room temperature in 20% methanol (diluted in ddH_2_O) for 30 min, 40% methanol for 30 min, 60% methanol for 30 min, 80% methanol for 30 min and 100% methanol for 30 min twice. The tissues were then bleached with 5% H_2_O_2_ (1 volume of 30% H_2_O_2_ diluted in 5 volumes of 100% methanol) containing 10 mmol/L EDTA (pH 8.0) at 4 °C for 48 h, and rehydrated at room temperature in 80% methanol (diluted in ddH_2_O) for 30 min, 60% methanol for 30 min, 40% methanol for 30 min, 20% methanol for 30 min and PBS/0.2% Triton X-100 for 1 h twice. The tissues were permeabilized in PBS/0.2% Triton X-100/20% DMSO/0.3 mol/L glycine at 37 °C for 24 h, and blocked in PBS/0.2% Triton X-100/10% DMSO/5% donkey serum (Jackson ImmunoResearch) at 37 °C for 24 h. The tissues were then incubated with indicated primary antibodies diluted (1:500-1:1,000) in PBS/0.2% Tween-20/10 μg/mL heparin/5% DMSO/5% donkey serum at 37 °C for 72 h, and washed in PBS/0.2% Tween-20/10 μg/mL heparin at 37 °C for 1 h five times. The tissues were incubated with indicated Alexa dye-conjugated secondary antibodies diluted (1:500) in PBS/0.2% Tween-20/10 μg/mL heparin/5% donkey serum at 37 °C for 72 h, and washed in PBS/0.2% Tween-20/10 μg/mL heparin at 37 °C for 2 h five times before the tissue clearing.

Immunolabeled iWAT was embedded in 1% agarose-blocks prepared in PBS. The tissue blocks were dehydrated in glass tubes at room temperature in 20% methanol (diluted in ddH_2_O) for 1 h, 40% methanol for 1 h, 60% methanol for 1 h, 80% methanol for 1 h, and 100% methanol for 1 h twice. The tissue blocks were incubated with the mixture of dichloromethane (Sigma)/methanol (2 volumes/1 volume) for 3 h, and then with 100% dichloromethane for 15 min twice. The tissue blocks were finally cleared with 100% dibenzyl-ether (Sigma) for 1 h twice to be ready for the volume fluorescence-imaging.

Optically-cleared iWAT was imaged on the LaVisionBiotec Ultramicroscope II equipped with six fixed lightsheet-generating lenses, the sCMOS camera (Andor Neo), and the 2×/NA0.5 objective (MVPLAPO) covered with the 6-mm working-distance dipping-cap. Version v144 of the Imspector Microscope Controller software supported by LaVisionBiotec was used. The tissue blocks were immersed in the chamber filled with 100% dibenzyl-ether for the volume-imaging procedure. For imaging at 1.26× effective magnification (0.63× zoom), the tissue blocks were scanned by the three combined lightsheets from the right side, with a step-size of 4 μm through each tissue block. For imaging at 12.6× effective magnification (6.3× zoom), the tissue blocks were scanned by the one single lightsheet (middle position) from the right side, with a step-size of 2 μm through each tissue block. The image stacks were acquired by the continuous lightsheet scanning method without the contrast-blending algorithm.

Imaris (http://www.bitplane.com/imaris/imaris) was used to reconstruct the image stacks obtained from the lightsheet imaging. To quantify the density of sympathetic arborizations, five 0.3 mm × 0.3 mm × 0.3 mm volumes were randomly selected from reconstructed 3D images of each iWAT, and lengths of the sympathetic fibers in each cubic volume were manually traced. For display purpose in the figures and movies, a gamma correction of 1.2–1.4 was applied onto the raw data. Movies of the image stacks were generated with the frame rate of 25 fps. 3D projections of the image stacks were generated with the orthogonal perspective for the representative images shown in figures.

### *In vitro* cultures

For the cultures of sympathetic neurons, the superior cervical ganglia were dissected from P1 neonatal wildtype mice. The ganglia were dissociated in 0.05% Trypsin/EDTA (Gibco) at 37 °C for 10 min. After washing once with Neurobasal/B27 medium (Neurobasal medium supplemented with 2% B27, 2 mmol/L glutamine, 100 U/mL penicillin, 100 μg/mL streptomycin, and 0.5% methylcellulose), the neurons were re-suspended in Neurobasal/B27 medium and seeded in 12-well plates coated with poly-*D*-lysine (Sigma) and Laminin (Life Technologies). To determine the NGF-stimulated axon outgrowth, recombinant mouse NGF (Sigma, final concentration of 25 ng/mL) or indicated conditioned-media (1:3 dilution) was added to the cultures for 48 h. Sympathetic neurons were fixed in PBS/1% PFA, immunostained with anti-tyrosine hydroxylase and corresponding Alexa dye-conjugated secondary antibody, and imaged by fluorescence microscopy.

For the *in vitro* treatment of iWAT, iWAT of *Adrb1*^−/−^; *Adrb2*^−/−^; *Adrb3*^−/−^ or control mice were acutely dissected out, washed twice in DMEM medium, and cut into small tissue pieces (approx. 2 mm × 2 mm × 2 mm). The tissues were then cultured in DMEM medium without or with norepinephrine for 6 to 12 h. The total RNAs were extracted by RNeasy Mini Lipid Tissue Kit, and processed for SYBR Green qPCR analysis. In parallel, the conditioned-media were collected and tested on cultured sympathetic neurons.

### Statistical method

Student’s two-sided *t*-tests were performed using GraphPad Prism (http://www.graphpad.com/scientific-software/prism). The statistical details of experiments can be found in the figure legends. No statistical methods were used to pre-determine the sample sizes.

## Electronic supplementary material

Below is the link to the electronic supplementary material.

Supplementary material 1 (MOV 11190 kb)

Supplementary material 2 (MOV 13752 kb)

Supplementary material 3 (PDF 273 kb)
