# Supplementary material for: Whole-tissue 3D imaging reveals intra-adipose sympathetic plasticity regulated by NGF-TrkA signal in cold-induced beiging
Source: Protein Cell. 2018 Mar 27;9(6):527–39. doi: 10.1007/s13238-018-0528-5 (PMC5966360; doi:10.1007/s13238-018-0528-5)
Supplement: Supplementary file 3 — Supplementary material 3 (PDF 273 kb) [file 13238_2018_528_MOESM3_ESM.pdf]

Supplemental Movie Legends

Movie S1. Volume fluorescence-imaging of sympathetic arborizations in iWAT of the wildtype mice maintained at room temperature, related to Figure 1.

The wildtype mice were maintained at room temperature, and their iWAT was processed for the whole-mount immunolabeling of tyrosine hydroxylase, and imaged at 12.6x magnification on the lightsheet microscope.

Movie S2. Volume fluorescence-imaging of sympathetic arborizations in iWAT of the wildtype mice after cold challenge, related to Figure 1.

The wildtype mice were subject to cold challenge for 5 days, and their iWAT was processed for the whole-mount immunolabeling of tyrosine hydroxylase, and imaged at 12.6x magnification on the lightsheet microscope.

Supplemental Figures and Legends

Figure S1

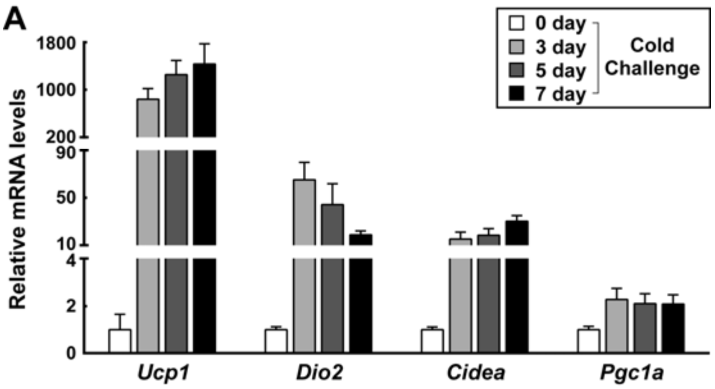

Figure S1. Intra-adipose sympathetic plasticity in response to cold challenge, related to Figure 1.

(A) The wildtype mice were subject to cold challenge. iWAT was harvested at indicated time points, and expression levels of the beiging-related genes were determined by the qPCR analysis. n=4, mean ± SEM.

Figure S2

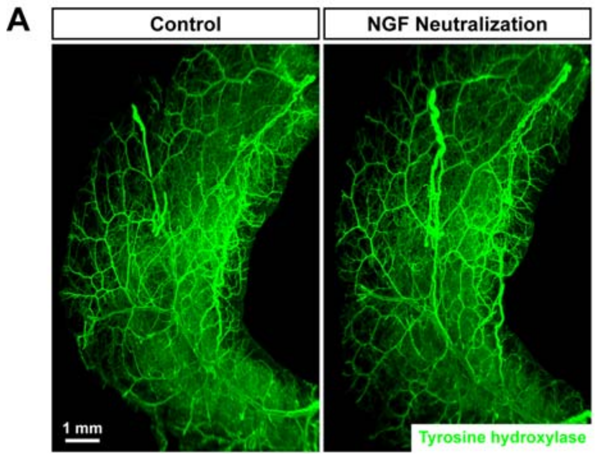

Figure S2. Intra-adipose sympathetic plasticity is regulated by NGF, related to Figure 4.

(A) The wildtype mice were administrated with NGF-neutralizing antibody or control IgG, and maintained at room temperature for 7 days. iWAT was harvested and processed for the volume fluorescence-imaging of anti-tyrosine hydroxylase. Representative 3D projections of iWAT imaged at 1.26x magnification on the lightsheet microscope.

Figure S3

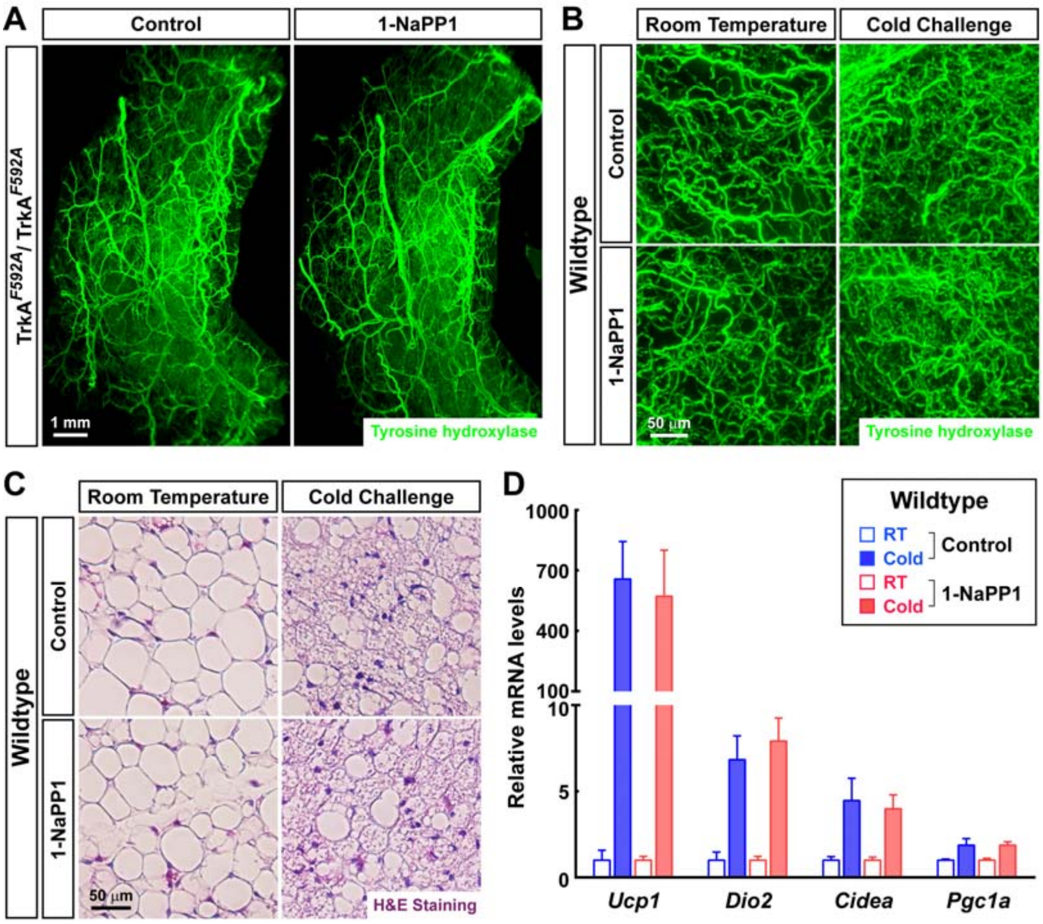

Figure S3. Intra-adipose sympathetic plasticity is regulated by the TrkA signal, related to Figure 5.

(A) *TrkA*$^{F592A/F592A}$ mice were daily treated with 1-NaPP1 or vehicle control. iWAT was processed for the volume fluorescence-imaging of anti-tyrosine hydroxylase. Representative 3D projections of iWAT imaged at 1.26x magnification on the lightsheet microscope. **(B to D)** The wildtype mice daily-treated with 1-NaPP1 or vehicle control were subject to cold challenge. **(B)** iWAT was processed for the volume fluorescence-imaging of anti-tyrosine hydroxylase. Representative 3D projections of iWAT imaged at 12.6x magnification on the lightsheet microscope. **(C)** Appearance of multilocular beige cells in iWAT was examined by H&E staining. **(D)** Expression levels of the beiging-related genes in iWAT were determined by the qPCR analysis. n=5, mean ± SEM.

Figure S4

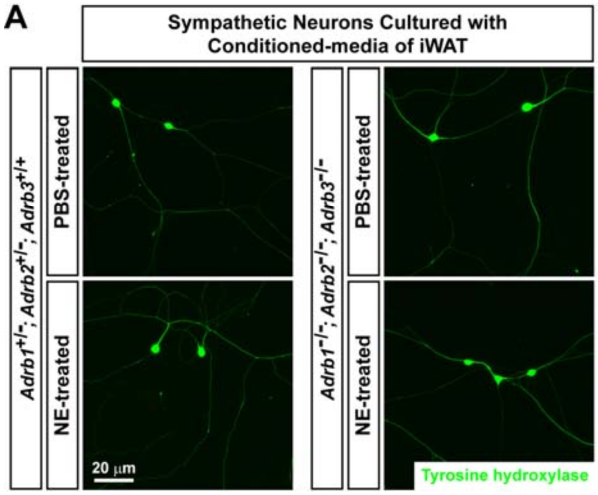

Figure S4. Cold-elicited NGF expression in WAT depends on the catecholamine signal, related to Figure 6.

(A) iWAT of *Adrb1*$^{-/-}$*; Adrb2*$^{-/-}$*; Adrb3*$^{-/-}$ mice or control mice (*Adrb1*$^{+/-}$*; Adrb2*$^{+/-}$*; Adrb3*$^{+/+}$) was *in vitro* treated with 100µM norepinephrine (NE) or control PBS. The conditioned-media of iWAT were collected and administrated to cultured sympathetic neurons. Representative images of sympathetic neurons immunolabeled by anti-tyrosine hydroxylase.
